# Development and Evaluation of a Droplet Digital PCR Assay for the Accurately Detecting the CircHIPK3 in Plasma Samples from Patients with Hepatocellular Carcinoma

**DOI:** 10.4014/jmb.2412.12048

**Published:** 2025-05-15

**Authors:** Yuanye Ji, Ping Tuo, Shun Zhang, Ting Cai, Liyun Fu, Qinzhi Deng, Houdao Fu, Guosheng Gao, Fajiu Wang, Peng Zhu

**Affiliations:** 1 Department of Medical Laboratory, Ningbo No.2 Hospital, Ningbo City, Zhejiang Province, P.R. China; 2 Ningbo Institute of Life and Health Industry, University of Chinese Academy of Sciences, Ningbo City, Zhejiang Province, P.R. China; 3 Department of Hepatology, Ningbo No.2 Hospital, Ningbo City, Zhejiang Province, P.R. China; 4 Department of Severe Hepatology, Ningbo No.2 Hospital, Ningbo City, Zhejiang Province, P.R. China; 5 Fu Houdao’s Traditional Chinese Medicine Expert Heritage Studio, Ningbo No.2 Hospital, Ningbo City, Zhejiang Province, P.R. China; 6 Department of Clinical Laboratory, Ningbo No.2 Hospital, Ningbo City, Zhejiang Province, P.R. China; 7 Department of Thoracic Surgery, Ningbo NO.2 Hospital, Ningbo City, Zhejiang Province, P.R. China

**Keywords:** ddPCR, circHIPK3, clinical sample detection, effect evaluation

## Abstract

Hepatocellular carcinoma (HCC) is an increasingly prevalent malignant neoplasm on a global scale. Circrna HIPK3 (circHIPK3) has been identified as playing a key role in HCC tumorigenesis and as a novel biomarker. In this study, we aimed at developing a sensitive and accurate method for the detection of circHIPK3 in low load plasma samples using a droplet digital PCR (ddPCR). We designed circHIPK3 gradient primers and probes and optimized the PCR system to improve performance. Then we assessed and compared the linearity and sensitivity of ddPCR and quantitative PCR (qPCR) using the circHIPK3 plasmid DNA as a template. Using these methods, circHIPK3 concentrations were quantitatively determined in 3 cell lines and 40 plasma samples to assess clinical stability. Within the plasmid concentration range of 3^1^-3^6^ copies/μl, the ddPCR exhibited a linear fitting equation of Y = 1.037X-0.1724 with *R^2^* value of 0.9940, which surpassed the corresponding *R^2^* value of 0.9877 for qPCR. Furthermore, the limit of blank (LOB) and limit of detection (LOD) for ddPCR were determined to be 0.157 copies/μl and 0.594 copies/μl, respectively, which were significantly lower than the LOD of qPCR (5.753 copies/μl). In clinical samples, ddPCR demonstrated a commendable correlation with qPCR, evidenced by a Kappa value of 0.677 (*p* < 0.05, 95% CI [0.503-0.851]) and an intraclass correlation coefficient (ICC) of 0.903 (95% CI [0.831-0.946]). Notably, ddPCR identified 11 positive samples that qPCR failed to detect. The ddPCR-based circHIPK3 liquid biopsy method emerged as a highly sensitive and accurate approach, lending itself well to clinical applications.

## Introduction

Hepatocellular carcinoma (HCC) is a common malignancy with a continuously increasing incidence worldwide [1]. The early detection of HCC currently relies on imaging and the levels of alpha-fetoprotein (AFP). Although imaging has high accuracy in the diagnosis of mid and late-stage HCC, its efficacy in the early detection of the disease is limited. Furthermore, the issue of false positives in AFP testing has restricted its widespread use in HCC screening, which is one of the critical factors contributing to the difficulty of early diagnosis and high mortality rate of HCC [2]. Recently, the detection of biomarkers based on liquid biopsy has become a focal point of research [3]. Among various biomarkers, circular RNA (circRNA) has emerged as a novel class of non-coding RNA that primarily regulates the expression of tumor-related gene through the sponge action of miRNAs, playing a pivotal role in the development and progression of various tumors [4]. The liquid biopsy based on circRNA provides a new strategy for the early biomarker detection in HCC. CircRNA HIPK3 (circHIPK3, circRNA ID: hsa_circ_0000284), composed of the second exon (1099nt) of the HIPK3 gene [5], promotes tumor metastasis and enhances the proliferation and invasion of cancer cells in HCC by acting as a sponge for miRNA [6-9]. Therefore, cicrHIPK3 holds potential to serve as a biomarker for the early detection of HCC, providing substantial value in the prevention and control strategies [5]. However, previous research on circHIPK3 has mainly focused on its biological mechanisms, while studies on its detection in low-concentration clinical liquid specimens, particularly in plasma samples from HCC patients, are relatively scarce. Thus, establishing a sensitive and accurate method for liquid biopsy is essential to facilitate the clinical application of circHIPK3 as a biomarker in HCC management. The prevalent methods for detecting circRNA include Northern blotting, qPCR, microarray analysis, and RNA sequencing [10]. Among them, Northern blotting, allowing to evaluate the presence and length of circRNA, is considered as the gold standard for analyzing circRNAs, yet it is hampered by poor sensitivity and lengthy processing time [11]. The qPCR, commonly used for quantifying the expression of cicrRNA, particularly in studies involving circHIPK3 mechanisms [7-9], tends to overestimate cicrRNA levels due to due to the potential formation of circular copies during the reverse transcription, which compromise its quantitative accuracy [12, 13]. RNA sequencing represents the most accurate method for circRNA detection. However, its application in routine diagnosis is constrained by the requirement of expensive reagents, sophisticated equipment, as well as trained data processing personnel [14, 15]. The data of circRNA detection produced by microarray-based approaches are inconsistent across different studies, which presents considerable challenges for comparative analysis [16]. In summary, these technologies are subject to practical limitations such as issues with accuracy, sensitivity and high costs. Therefore, these technologies are more suitable as screening tools rather than for quantitative analysis. Droplet Digital PCR (ddPCR) represents a third-generation PCR, and its quantification approach diverges from that of traditional PCR [17]. This technique employs a "water-in-oil" emulsion or microfluidic chips to partition the PCR mixture into droplets. These droplets, each containing nucleic acid molecules, are randomly distributed into more than 10000 independent reaction chambers, ensuring the presence of nucleic acid molecules in each chamber. Subsequently, the target gene within each chamber undergoes PCR amplification [18]. After amplification, the analyzing instrument collects fluorescence signals from all chambers. By setting a threshold for these signals and employing Poisson distribution calculations, the copy number of the target gene in each sample is determined [19]. Compared to qPCR, ddPCR offers absolute quantification of target DNA, circumventing the need for a standard curve and addressing low accuracy of qPCR when quantifying circRNAs [20]. With its enhanced sensitivity, ddPCR is adept at detecting low DNA copy number variations in samples, making it especially suitable for low copy number detection [21]. Furthermore, the droplet generation process mitigates common issues encountered in traditional nucleic acid quantification, such as the instability of weak positive results [22]. This process also ensures consistent amplification efficiency in each individual reaction chamber, eliminating the influence of inhibitors on amplification [23]. Given these substantial benefits, ddPCR is expected to become a pivotal tool for the quantitative analysis and detection of circRNAs [24-27].

**Fig. 1:**
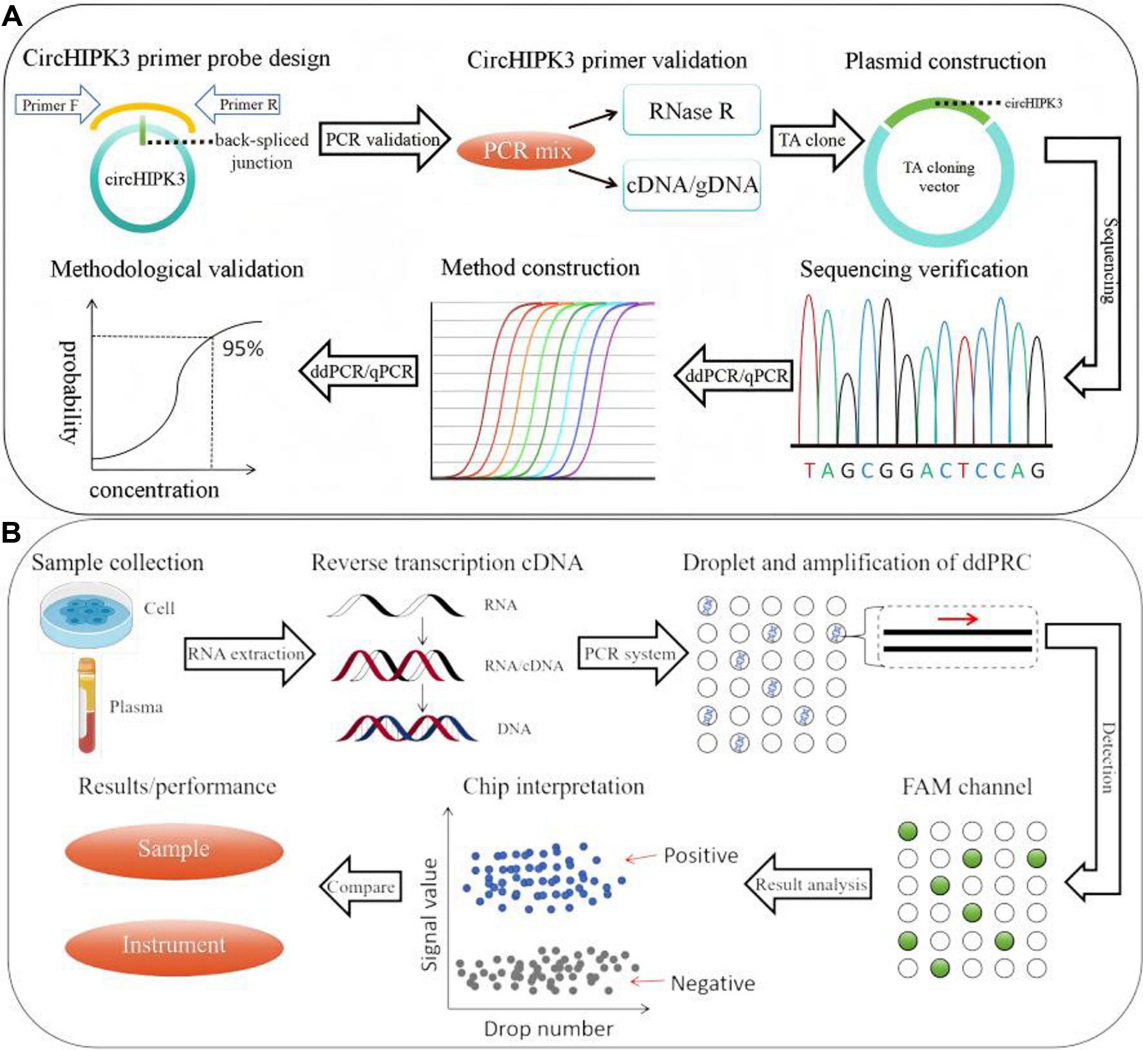
Experimental procedure. (**A**) ddPCR circHIPK3 detection method construction process. The construction of detection method includes 6 steps: primer design, primer validation, plasmid construction, sequencing validation, PCR system optimization and methodology validation. Primer design follows primer design principles. CircHIPK3 requires the design of div primers and con primers for subsequent validation, where div primers need to contain or cross back-spliced junction sites. Primer validation was carried out by the RNase R experiment and cDNA/gDNA comparison experiment. Plasmid construction was verified by Sanger sequencing. PCR system optimization included primer probe combination and annealing temperature screening. Methodology validation included linear fitting and sensitivity comparison of ddPCR and qPCR. (**B**) ddPCR circHIPK3 detection method to detect cells and clinical plasma sample flow. ddPCR clinical sample testing consists of five steps: RNA extraction, reverse transcription of cDNA, droplet generation, amplification, and analysis. PCR system will be dispersed in more than 10000 microchambers after droplet transformation, and the droplets in all microchambers will undergo completely independent PCR amplification reaction, minimizing other interfering substances that may exist in the reaction system from a physical level. The absolute quantification of nucleic acid copy number is realized by Poisson distribution analysis of negative and positive signals. In addition, the results of two different samples were analyzed after ddPCR detection was completed; at the same time, the ddPCR detection results were compared with the qPCR detection results performed simultaneously to evaluate the applicability of ddPCR in sample detection.

In this study, we established a ddPCR-based circRNA liquid biopsy for detecting circHIPK3 in clinical samples of HCC, illustrated in Fig. 1A. We optimized the assay for precision, comparing its sensitivity and reliability to qPCR in cellular and clinical matrices. Our results indicated that the ddPCR method has superior sensitivity and accuracy for detecting low copy circHIPK3, suggesting its potential as a reliable alternative to qPCR in the liquid biopsy of circRNA biomarkers in HCC clinical samples.

## Methods

### Reagents

TransZol Up Plus RNA Kit, TransScript All-in-One First-Strand cDNA Synthesis SuperMix for qPCR (One-Step gDNA Removal) and pEASY-T1 Cloning Kit were purchased from TRANS (China). DEPC H_2_O was purchased from Biosharp (China). Premix Ex Taq (Probe qPCR) and Hind III were purchased from TaKaRa (Japan). Fetal bovine serum (FBS) and DMEM medium were purchased from Gibco BRL Inc. (USA). Table 1 lists all the primer, probes and plasmid sequences used in this study. These primers and probes were synthesized by Shenggong Company (China) and dissolved in DEPC H_2_O and stored at -20°C for use in subsequent experiments.

### Blood Sample Processing

We collected 25 whole blood samples from HCC patients and 15 from healthy individuals at Ningbo No.2 Hospital (Table S1). Whole blood was collected using K2EDTA vacuum venous tubes with an inert gel barrier. Within 2 h of sample collection, plasma was isolated by centrifugation at 3,000 rpm for 10 min at 25°C, effectively removing the majority of blood cells. The resulting plasma was aliquoted into 800 μl and stored at -80°C for subsequent experiments [28]. The use of clinical samples was approved by the Ethics Committee of Ningbo No.2 Hospital.

### Cell Culture

HepG2, Huh7, and LX-2 cell line purchased from China Center for Type Culture Collection (CCTCC, China) was selected and inoculated into a 10 cm culture dish at a concentration of 2 × 10^5^ cells/ml. The cell line was cultured in DMEM medium containing 10% fetal bovine serum (FBS) and placed in an incubator at 37°C and cultured in 5% CO_2_ for 24 h.

### Plasmid Construction

To optimize the PCR system and validate the sensitivity and repeatability of the method, a plasmid was constructed using the pEASY-T1 cloning vector, according to the user manual. The cloning sequence was presented in Fig. S1. The plasmid was linearized using Hind III and then subjected to a 3-fold gradient dilution. The copy number of plasmid DNA in the dilution series was calculated according to the following formula (1). In this formula, *E* is the copy of plasmid DNA dilution solution (unit: copies/μl) C is the concentration of plasmid DNA dilution solution (unit: g/ml), MW is the average molecular weight of double stranded DNA (unit: g/mol), and NA is the Avogadro constant (6.02 × 10^23^).

$$E=\frac{C}{MW}\times NA$$ (1)

### Primer and Probe Design

**Fig. 2:**
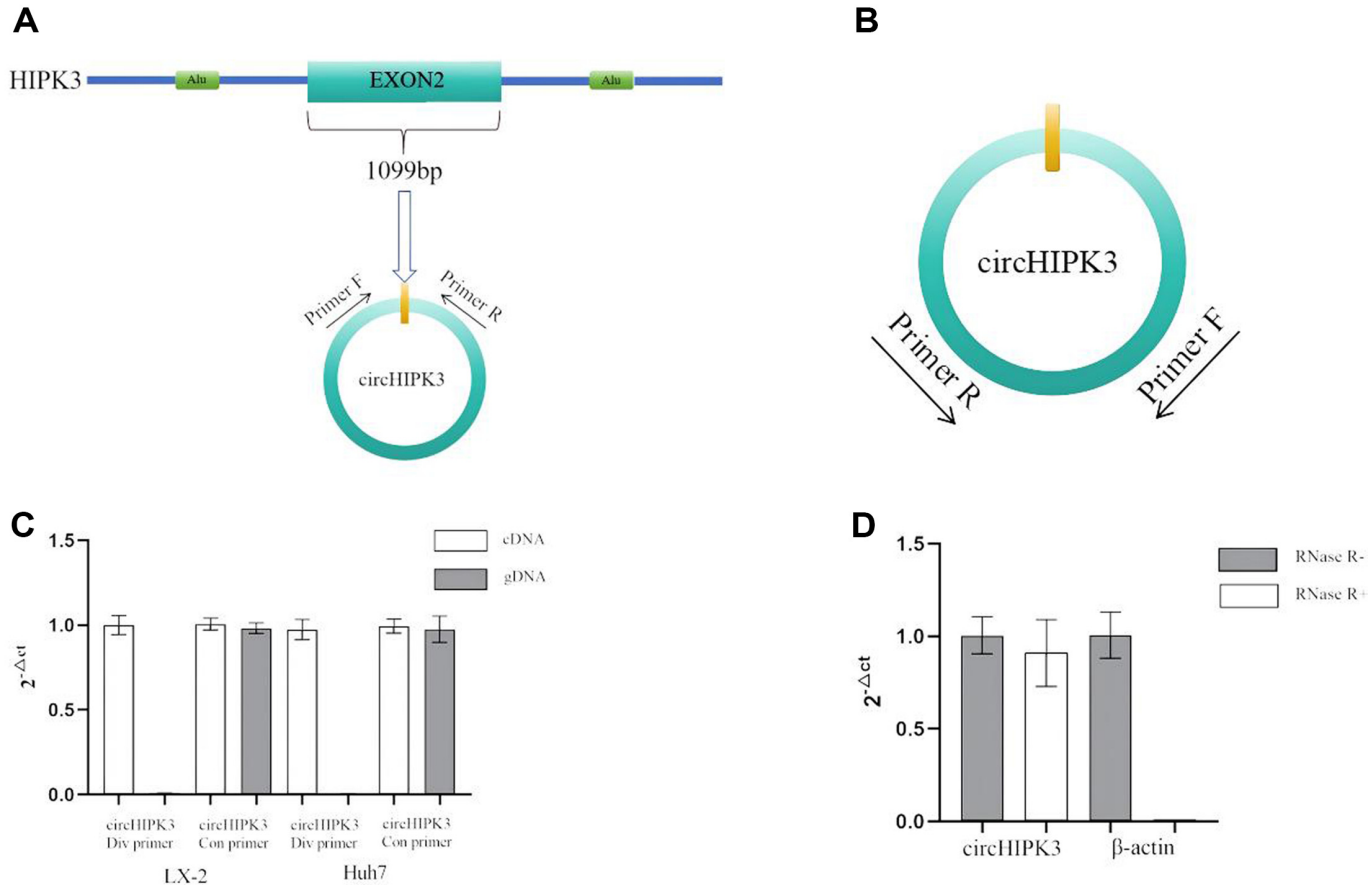
Primer design and validation. (**A**) The formation of circHIPK3 and the design location of the diverting prime. CircHIPK3 is composed of the second exon of the HIPK3 gene, with a total length of 1099bp and long introns on both sides. The multiple short repetitive sequences between the exon and intron are important reasons for the formation of circHIPK3. The div primers required to construct the detection method should be designed on both sides of the circHIPK3 back spliced junction site. (**B**) The results of the cDNA/gDNA experiment at the design position. (**C**) Amplification of circHIPK3 primers in different liver associated cell nucleic acids. Div primers can only amplify circHIPK3 in cDNA and cannot amplify in gDNA, the internal reference gene was β-Actin. (**D**) RNase R experimental results. The amplification of circHIPK3 by div primers is not affected by RNase R digestion. Linear amplification of the β-actin gene is degraded by RNase R.

Primers and probes were designed using Primer Premier 5 software. The original gene sequence was obtained from NCBI (GenBank: NM_005734.5:510-1608). The design criteria were as follows: (a) primer and probe bases should have a GC content ranging between 40%-60%, with bases distributed randomly; (b) Divergent primers (div primers) for circRNA should be designed to include or span back-spliced junction sites (Fig. 2A), and convergent primers (con primers) should be designed according to normal primer design (Fig. 2B); (c) the first base at the 5'end of the probe should not be G. The fluorescence signal of the probe label was 5'-6-FAM/3'-MGB. To verify whether the designed circHIPK3 primer/probes were available, experiments were conducted involving PCR amplification of cDNA/gDNA from cell samples, along with RNase R degradation assays. The qPCR results were calculated according to Eq. (2) [31].

$$2^{-\Delta ct}=2^{-\left(CT_{T\arg et}-CT_{reference}\right)}$$ (2)

### DdPCR Detection

**Fig. 3:**
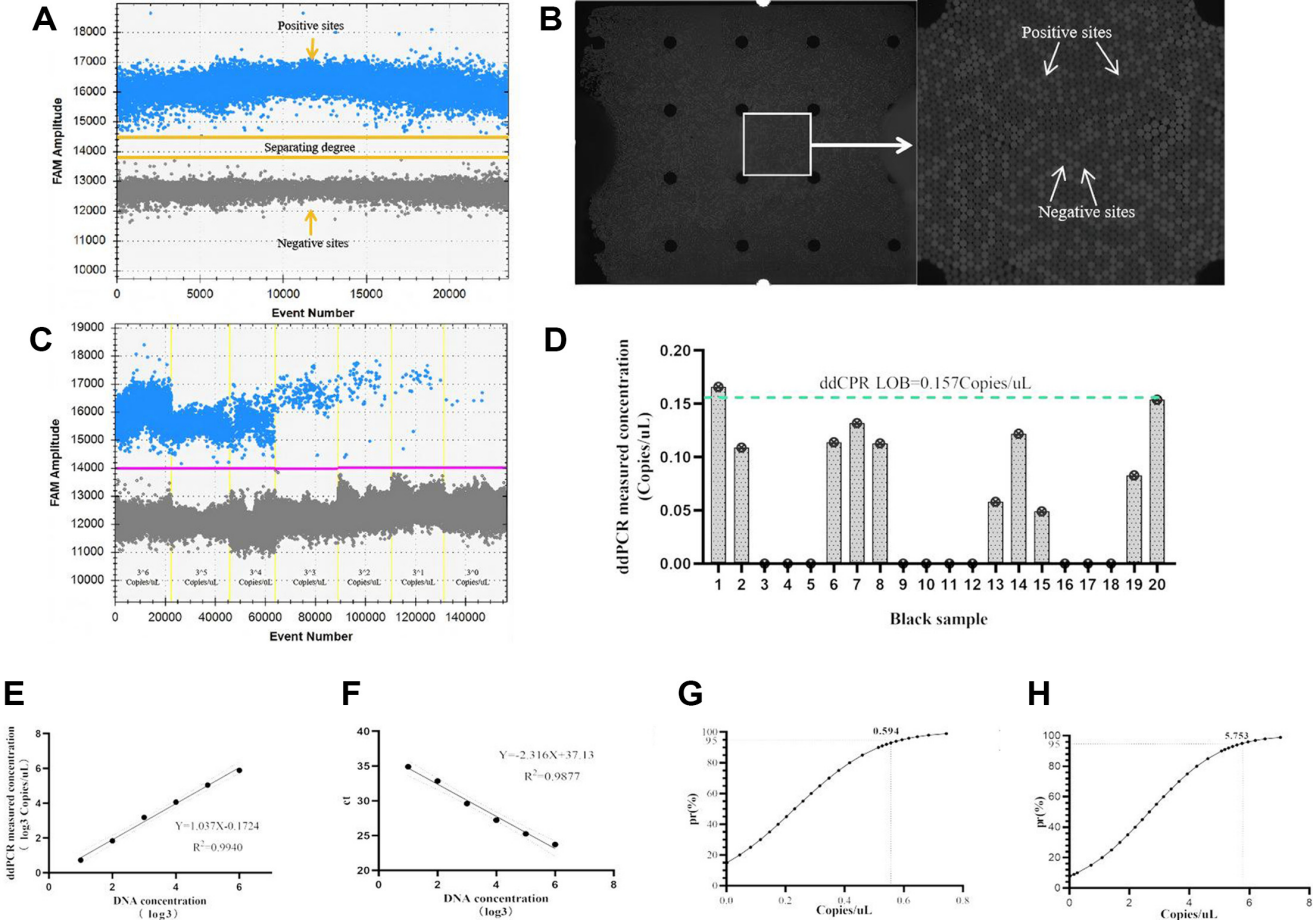
Construction of ddPCR detection method. (**A**) ddPCR result analysis interface, blue dots represent positive droplets, gray represents negative droplets. (**B**) Chip photo, positive droplets can detect fluorescent signals. Negative droplets do not detect fluorescent signals. (**C**) ddPCR results for 20 blank samples. LOB: Blank limits. (**D**) ddPCR concentration gradient analysis results. Range: 3^1^-3^6^ copies/μl. (**E**) ddPCR concentration gradient regression curve. Regression curve range: 3^1^-3^6^ copies/μl. (**F**) qPCR concentration gradient regression curve. Regression curve range: 3^1^-3^6^ copies/μl. (**G**) ddPCR LOD curve, 95%pr represents LOD, LOD = 0.594 copies/μl. H: LOD curve of qPCR method, 95%pr represents LOD, LOD = 5.753 copies/μl.

ddPCR detection was performed on the D3200 droplet digital PCR System (Pilot, China), with a total reaction volume of 14 μl, consisting of 7 μl Premix Ex Taq (Probe qPCR), 1.4 μl cDNA template, DEPC H_2_O, primer and probe. In order to improve the accuracy and stability of the detection system, the annealing temperature and the combination of primer and probe concentration were optimized. Owing to budgetary consideration, the system optimization was conducted on qPCR platform. Five distinct annealing temperatures (56°C, 58°C, 60°C, 62°C, 64°C) and twenty different combinations of primer and probe concentrations (ranging from 0.2-0.6 μM for 10 μM PCR primer-F/R and 0.1-0.5 μM for 10 μM PCR probes) were tested to determine the most favorable conditions for subsequent assays. Amplification conditions remained unchanged except for the parameters that needed to be optimized. Each optimization experiment was repeated three times to ensure the robustness of the optimization data. The specific reaction sequence was as follows: 95°C for 30 s (pre-denaturation), followed by 40 cycles of 95°C for 5 s (denaturation), annealing and extension for 30 s, then cooled to 50°C for 15 s. The final analysis interface is shown in Fig. 3A.

### Performance Evaluation and Method Comparison

To investigate the sensitivity of the ddPCR detection method, the threefold gradient plasmid diluted plasmid solution (with concentration ranging from 3^1^-3^6^ copies/μl) was used to perform sensitivity assays. Each concentration level was tested in triplicate. And synchronous experiments were conducted on the qPCR platform for comparative analysis. A regression analysis of the results was conducted to evaluate the disparities in detection capabilities between the two PCR platforms. The *R^2^* parameter was used to quantify the linear fit of PCR methods. The blank limit (LOB) of the ddPCR method was calculated according to formula (3) [29, 30], where the average value of the blank sample results (in copies/μl is the unit), SD is the standard deviation of the blank sample result (in copies/μl is the unit) of measurement. A total of 20 blank samples (DEPC H_2_O) were detected. The limit of detection (LOD) is defined as the minimum concentration at which a method can detect 95% positive results [32].

$$LOB=\bar{X}+1.645SD$$ (3)

### Sample Detection

A total of 3 different cell lines (HepG2, Huh7 and LX-2) and 40 clinical plasma samples (25 from HCC patients and 15 from healthy individuals) were used to verify the applicability of ddPCR method in clinical samples. Total RNA from samples was extracted using the TransZol Up Plus RNA Kit according to the user manual, eluted in 30 μl DEPC H_2_O, and analyzed for quality using a Nanodrop ND-1000 spectrophotometer (Thermo Scientific, USA). Total RNA was then reverse transcribed into cDNA by TransScript All-in-One First-Strand cDNA Synthesis SuperMix for qPCR (One-Step gDNA Removal). ddPCR testing was performed in triplicate for each sample. Experiments were performed simultaneously on the qPCR platform for subsequent comparative analysis.

### Data and Statistical Analysis

The ddPCR data were analyzed using Gene PMS software (Pilot Gene, China). The exclusion criteria for the data included: (a) an insufficient number of acceptable droplets, less than 12000 droplets; and (b) an assay concentration falling below the LOB. The qPCR data were analyzed via qPCR soft 4.1 software (Analutik Jena, Germany). Samples above 36 Ct are considered false positive [33]. Kappa coefficient analysis, Intraclass Correlation Coefficient (ICC) analysis were performed by IBM SPSS Statistics 23 software. A *p*-value of less than 0.05 was considered to indicate statistical significance. Sensitivity calibration curve analysis and T test were performed with Graph Pad Prism 8.0 software, again with *p* < 0.05 denoting statistical significance. All graphs were drawn using IBM SPSS Statistics 23, Graph PadPrism 8.0 and PowerPoint software. Additionally, centain graphical material was sourced from SCIDRAW and Medpeer websites, enhancing the visual documentation of the research findings.

## Results

### Primer Validation

The primers for the detection of circHIPK3 were designed in accordance with circRNA primer design principles [34], which were based on the gene sequence retrieved from the NCBI database. To authenticate these primers, two distinct verification methods were implemented. The first method involved PCR amplification of cDNA/gDNA from cell samples, wherein div primers positioned on either side of the back-spliced junction site were capable of amplifying circRNA from cDNA. Conversely, these primers, when oriented in reverse direction in gDNA, failed to facilitate amplification. While the con primers were able to amplify both cDNA and gDNA across various cells (Fig. 2C). The second method entailed an RNase R degradation assay. After RNase R treatment, the linear RNA within the total RNA from cell samples is substantially degraded. Owing to its closed loop structure, circHIPK3 is notably resistant to degradation by RNase R. The cicrHIPK3 primer continued to perform normal amplification, whereas the linear RNA-based β-actin gene could not be amplified (Fig. 2D). These outcomes confirmed the suitability of the div primer for circHIPK3, thus validating its use for further experimental procedures.

### System Optimization

The optimal amplification conditions are pivotal in enhancing the accuracy of PCR-based methods. Given the similarities between the optimal amplification conditions for ddPCR and qPCR, the system optimization was conducted on qPCR platform to economize on expenses. Initially, the ideal annealing temperature in the detection process was determined by comparing Ct values of different annealing temperatures (56°C, 58°C, 60°C, 62°C, 64°C). As shown in Fig. S2, the lowest Ct value was observed at an annealing temperature of 58°C, which was designated as the optimum temperature for subsequent experiments. Then, various combinations of primer and probe concentration (10 μM PCR primer-F/R 0.2-0.6 μM, 10 μM PCR probe 0.1-0.5 μM) were evaluated to identify the ideal combination, also based on the Ct value. Fig. S3 indicated that Ct value was minimized at a combination of 0.5 μM primer-F/R and 0.5 μM probe. Consequently, this combination was selected for ensuring experiments.

### Sensitivity Detection

The sensitivity of ddPCR was demonstrated through the examination of a gradient-diluted plasmid solution, with findings presented in Fig. 3C. The ddPCR method reliably detects circHIPK3 in concentrations ranging from 0.8 to 1 copies/μl. Additionally, twenty blank samples (DEPC H_2_O) were assessed, and the limit of blank (LOB) for the ddPCR method was calculated to be 0.157 copies/μl, following Eq. (2) as shown in Fig. 3D. For comparison purposes, three-fold dilutions of plasmid solutions were also tested on the qPCR platform. The results for both ddPCR and qPCR platforms were individually represented in Fig. 3E and 3F. A robust linear relationship was observed between plasmid concentration (log3) and signal output on both platforms, within a plasmid concentration range of 3^1^-3^6^ copies/μl. The fitting equations were established as ddPCR: Y = 1.037X-0.1724, with an *R^2^* of 0.9940; qPCR: Y = -2.316X +37.13, with an *R^2^* of 0.9877, indicating that ddPCR exhibited a higher degree of linear fitting than qPCR. The limits of detection (LOD) of ddPCR and qPCR platforms were identified to be 0.594 copies/μl and 5.753 copies/μl, respectively, as shown in Fig. 3G and 3H. Notably, the LOD of ddPCR platform was significantly more sensitive than the qPCR platform.

### Evaluation of Cellular and Clinical Plasma Samples

**Fig. 4:**
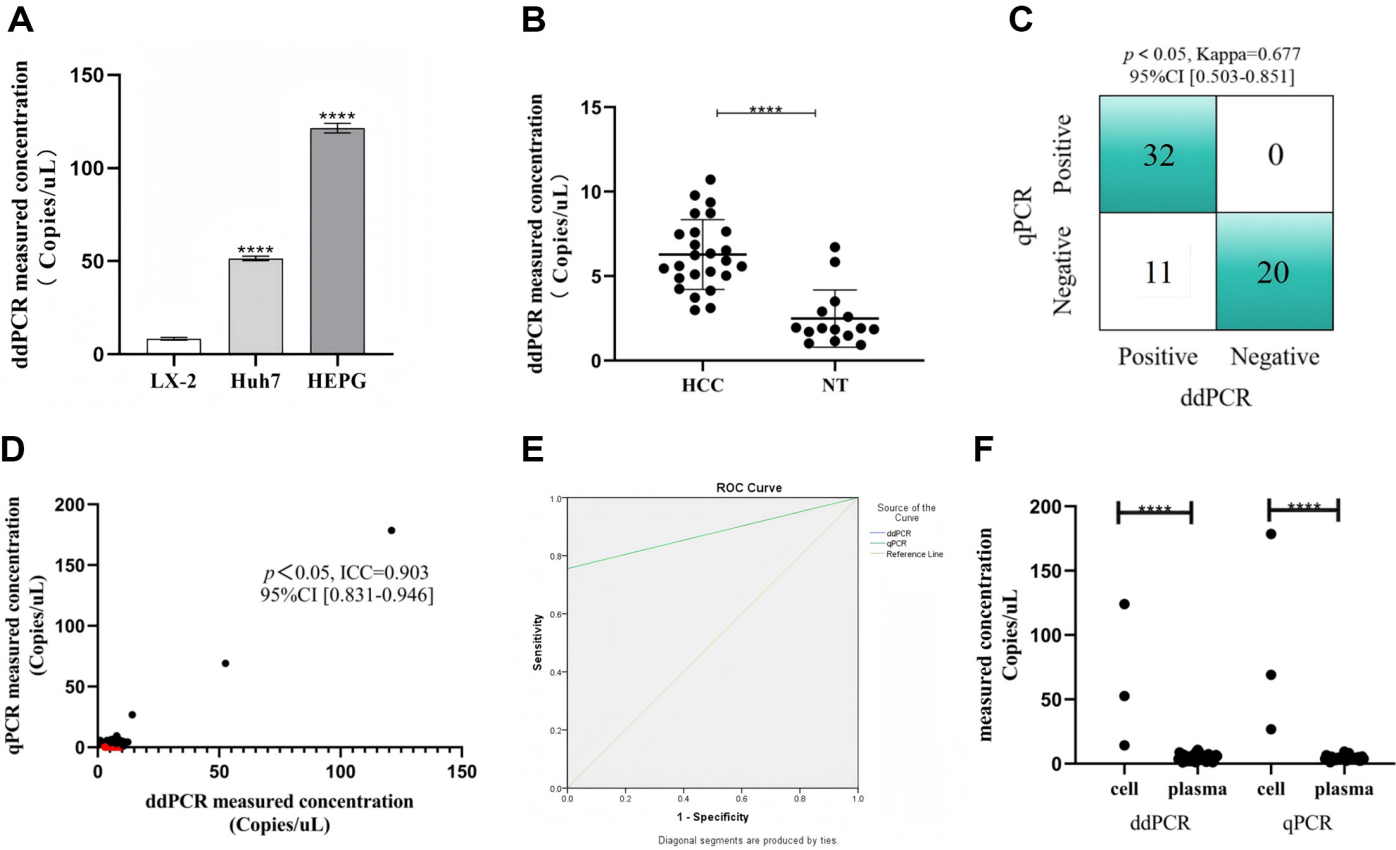
Clinical sample results and instrument comparison analysis. (**A**) ddPCR detection of healthy cells LX-2 and liver cancer cells Huh7, HEPG results, *p* < 0.01. (**B**) ddPCR results of healthy blood samples and HCC plasma samples, *p* < 0.01. (**C**) Summary of positive, blank (negative) sample results. Kappa = 0.677,95%CI [0.503-0.851]. (**D**) Correlation analysis between ddPCR and qPCR results of 39 samples. ICC = 0.903,95%CI [0.831-0.946]. The red dots are samples detected by ddPCR but not by qPCR. (**E**) ROC curve. The ddPCR results were used as the reference. qPCR AUC = 0.878, using positive and negative samples as references. (**F**) Comparison of test results of cell samples and plasma samples.

In order to clarify the applicability of ddPCR detection method in actual samples, an evaluation was performed on three types of cells and 40 plasma samples from Ningbo No.2 Hospital, as depicted in Fig. 1B. The ddPCR could detect circHIPK3 in all tested samples, indicating that the ddPCR method established in this study could accurately quantify circHIPK3 across diverse sample types. Furthermore, a notable elevation in circHIPK3 expression was observed in HepG2 and Huh7 liver cancer cells compared to the normal liver cells LX-2, with statistical significance (*p* < 0.01), as illustrated in Fig. 4A. Parallel findings were mirrored in clinical plasma samples. The expression level of circHIPK3 in the plasma samples of HCC patients was significantly higher than that in the plasma of healthy individuals (*p* < 0.01), as shown in Fig. 4B. These results indicated that circHIPK3 is consistently upregulated in both the cells and plasma of HCC patients, which affirmed previous studies [7-9]. To compared with the qPCR platform, cell and plasma samples were simultaneously subjected to analysis using qPCR. Subsequent assessments, including Kappa coefficient analysis, ICC analysis, and ROC analysis, were conducted on these two sets of results. Fig. 4C depicts the ddPCR and qPCR outcomes for positive and negative samples, with the Kappa coefficient analysis indicating that ddPCR and qPCR have good consistency in the qualitative analysis of circHIPK3 (Kappa = 0.677, *p* < 0.05, 95% CI [0.503-0.851]). However, compared to ddPCR, qPCR detected 11 fewer positives among 43 tested samples. The ICC analysis results, shown in Fig. 4D, revealed a strong correlation between ddPCR and qPCR detection results (ICC = 0.903, 95% CI [0.831-0.946]). The ROC curve, displayed in Fig. 4E, since all positive samples were detected by ddPCR, while the AUC for qPCR, using ddPCR results as a reference, was 0.878. Nonetheless, when plasma samples were analyzed by qPCR, the results for HCC samples were significantly different from those of healthy controls (*p* < 0.05, Fig. S4). This was less pronounced compared to the ddPCR results (*p* < 0.01), which may be attributed to the higher sensitivity of ddPCR. This indicated that while both methods exhibit commendable concordance in detecting circHIPK3 in clinical samples, ddPCR demonstrated superior detection capabilities. Finally, a comparison of cell and plasma samples detecton on both ddPCR and qPCR platforms, as shown in Fig. 4F, revealed the content of circHIPK3 in cell samples were higher than in plasma samples across both platforms. However, qPCR detected a higher circHIPK3 content in cell samples compared to ddPCR. This discrepancy could be attributed to the propensity of circular RNA to form circular copies during reverse transcription, which often leads to an overestimation of circRNA concentrations when quantified by qPCR [10], thereby reducing the accuracy of the detection. Additionally, the AFP levels and circHIPK3 expression levels of plasma samples were compared with the average values from healthy samples. If the expression level was higher than the average value of healthy samples, it was considered positive; otherwise, it was considered negative. These data were then analyzed using the Kappa statistic. The results showed a high consistency between plasma AFP levels and circHIPK3 expression levels (Kappa = 0.652, 95% CI [0.411–0.893]). This suggests that circHIPK3 is associated with a clinical biomarker, highlighting its potential relevance in clinical diagnosis.

## Discussion

CircRNAs have been identified as potential miRNA sponges, exerting influence on diseases such as cancer; however, their linear sequences do not inherently possess this capacity [35, 36]. Therefore, at the stage of primer and probe design, it is pivotal that special div primers are crafted to ensure the amplification of circular RNA rather than linear RNA [34]. To conclude, in this study, div primers spanning the back spliced junction site of circHIPK3 were designed, and the specificity of the amplified product as circHIPK3 was confirmed through sequencing validation. In the aspect probe design, due to the short length of the PCR amplification sequences that were designed, there are limited sites available for probe design, resulting in probes with lower annealing temperatures. To avoid compromising the detection efficiency, an MGB modification was added to the 3 'end of the probe, which enhanced the fluorescence reporting efficiency of the probe in the study. In pursuit of elevating the amplification efficiency, the concentration combination of primer and probe and annealing temperature within the PCR system were optimized using the constructed circHIPK3 plasmid as a benchmark. After optimization, it was discerned that the ddPCR method exhibited higher sensitivity and accuracy compared to the qPCR method under identical conditions, which proved that the ddPCR method developed in this study was able to replace qPCR for more accurate quantitative analysis of circRNA.

In the assessment of actual samples, a comparative analysis was conducted between the ddPCR and qPCR methods in their application to cell and plasma samples. The results of the study demonstrated a notable superiority of ddPCR over qPCR in accuracy and sensitivity. The ddPCR method was observed to yield greater accuracy in cell samples detection and exhibited enhanced sensitivity when analyzing plasma samples with low concentrations. These results implied that ddPCR, through its absolute quantification capability, could address the quantification challenges associated with circRNA circular copies generated during reverse transcription, a limitation often encountered with qPCR. Owing to its heightened sensitivity, ddPCR was especially advantageous for the detection of circRNAs in liquid biopsy samples where low concentrations are prevalent. Moreover, this study identified the differential expression of circHIPK3 in plasma samples between HCC patients and healthy individuals, revealing the levels of circHIPK3 in the plasma of HCC patients are indeed higher than those in the healthy group. Nevertheless, the present study employed this finding merely as an instance to validate the application of ddPCR in the detection of clinical samples and did not delve into its diagnostic value, such as establishing specific thresholds for circHIPK3 levels in HCC patients versus healthy individuals or investigating the relationship between circHIPK3 expression and HCC progression and across various developmental stages. Therefore, it is posited that future research should be explored to the diagnostic and prognostic implications of circHIPK3 in HCC. Additionally, analysis of both cell and plasma samples revealed that the expression level of circHIPK3 was higher in cell samples than in plasma samples. This discrepancy may be due to differences in total RNA content between the two sample types or the preferential enrichment of circHIPK3 with cells. However, the underlying mechanisms behind this variation remain to be elucidated.

**Fig. 5:**
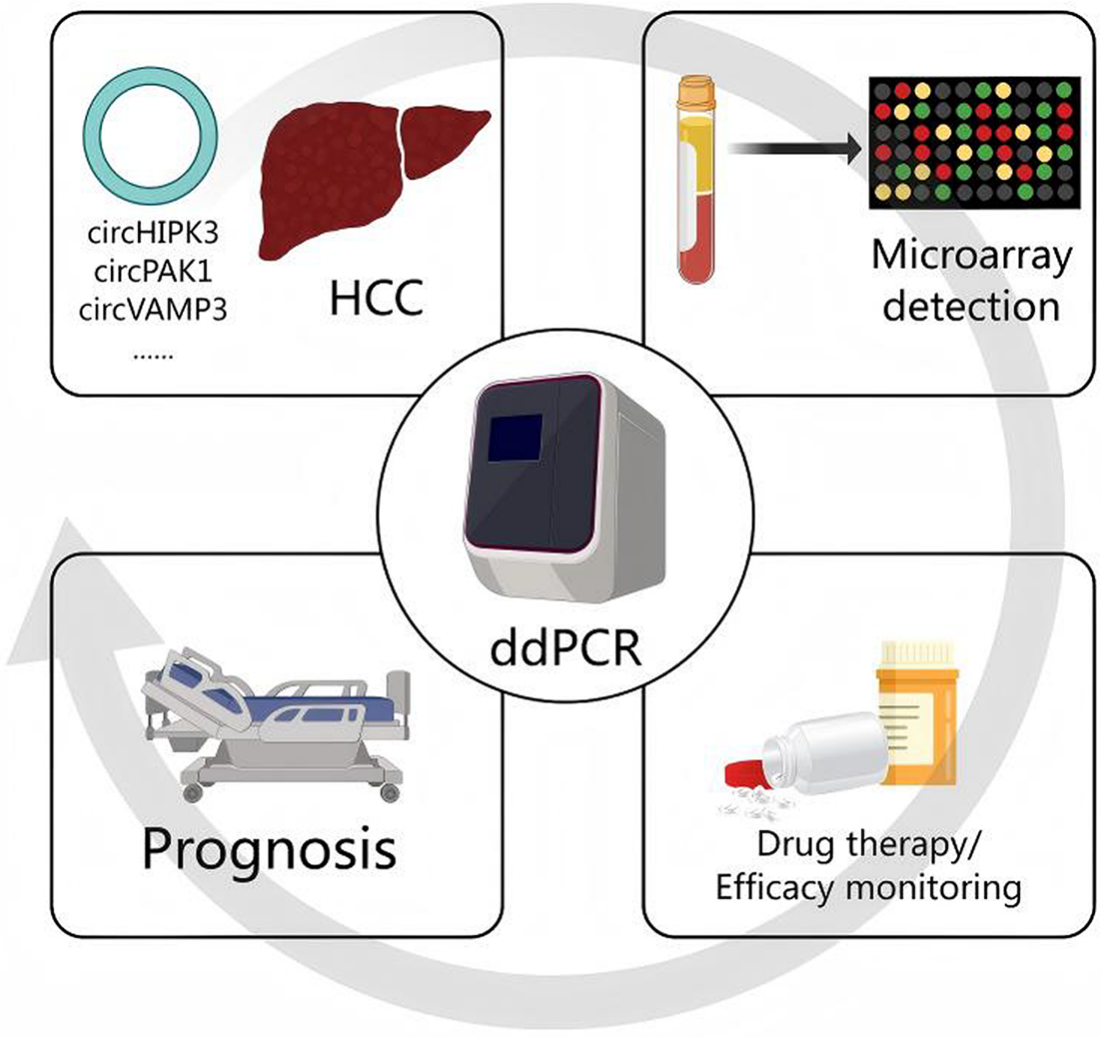
Clinical application direction of ddPCR. ddPCR can be used to detect circRNA that has been demonstrated to be a biomarker for HCC. Detection of circRNA in plasma and other liquid samples by microarray plays an important role in early diagnosis, monitoring of efficacy and prognosis.

Another significant advantage of ddPCR is that it serves as an extension of the PCR methodology, allowing for the optimization of amplification conditions on more cost-effective qPCR instruments. This means that those assays for detecting cicrRNA, initially conceived for qPCR, could be readily transitioned to ddPCR platforms, thereby achieving greater detection accuracy. In recent years, a number of studies focusing on cicrRNA in HCC, such as circRNAs ZKSCAN1 [37], circPAK1 [38], circGPR137B [39] and circVAMP3 [40], have been reported in prominent academic publications. If these circRNA biomarkers could be integrated and developed into a microarray detection method, it would significantly advance the use of ddPCR techniques in the clinical setting over RNA sequencing, becoming the preferred method for the quantitative detection of cicrRNA biomarkers. This would not only aid in the early diagnosis of HCC but also play a pivotal role in monitoring treatment efficacy and prognosis (Fig. 5). Therefore, the pursuit of developing a multiplex ddPCR liquid biopsy method capable of simultaneously detecting multiple HCC circRNA biomarkers, based on ddPCR technology, remains our future research goal.

## Conclusion

This study successfully developed a circHIPK3 detection method based on the ddPCR platform for the liquid biopsy of HCC, exhibiting sensitivity and accuracy superior to traditional qPCR. The significant advantages of ddPCR in accuracy and sensitivity offer a more reliable tool for detecting trace amounts of circRNA in clinical samples, particularly in differentiating circHIPK3 expression levels between HCC patients and healthy controls, indicating its potential clinical application. The findings underscore the importance of ddPCR in future clinical diagnostics and disease monitoring and lay the foundation for extending to a broader range of circRNA biomarker detection.

## Supplemental Materials

Supplementary data for this paper are available on-line only at http://jmb.or.kr.



## Figures and Tables

**Table 1:** Primers, probes, and plasmid sequences used in the study.

Name	Sequence (5'-3')	Length
COPY-F	GTTCAACATATCTACAATCTC	21 bp
COPY-R	ATTATCCAACTCCTCACT	18 bp
Div primer-F	TCGGTACTACAGGTATGGCCT	21 bp
Div primer-R	AGTAGAGCCAAGCAGTTGTGT	21 bp
Div probe	FAM-TTTATCAAACTCAGTCAAGTGCCTTT-MGB	26 bp
Con primer-F	GTCATAGCAGCTCAGGCACA	20 bp
Con primer-R	ACTCCTCACTCTTGCGCTTC	20 bp
Con probe	FAM-TCACGTGCAGGCACCTCAGATTGG-MGB	24 bp
Actin-F	GGGCATGGGTCAGAAGGATT	20 bp
Actin-R	ACCCTGAAGTACCCCATCGA	20 bp
Actin-P	FAM-CCCAGAGCAAGAGAGGCATC-MGB	20 bp
circHIPK3 plasmid	......TCCACGGACCTATGTGAATGGTAGAAACTTTGGAAATTCTCATCCTCCCACT AAGGGTAGTGCTTTTCAGACAAAGATACCATTTAATAGACCTCGAGGACACAAC TTTTCATTGCAGACAAGTGCTGTTGTTTTGAAAAACACTGCAGGTGCTACAAA GGTCATAG......(Partial fragment)	441 bp
