# Supplementary material for: Development and Evaluation of a Droplet Digital PCR Assay for the Accurately Detecting the CircHIPK3 in Plasma Samples from Patients with Hepatocellular Carcinoma
Source: J Microbiol Biotechnol. 2025 May 15;35:e2412048. doi: 10.4014/jmb.2412.12048 (PMC12099620; doi:10.4014/jmb.2412.12048)
Supplement: Supplementary file 1 [file jmb-35-e2412048-supple.pdf]

J. Microbiol. Biotechnol. 2025. 35: e2412048

https://doi.org/10.4014/jmb.2412.12048

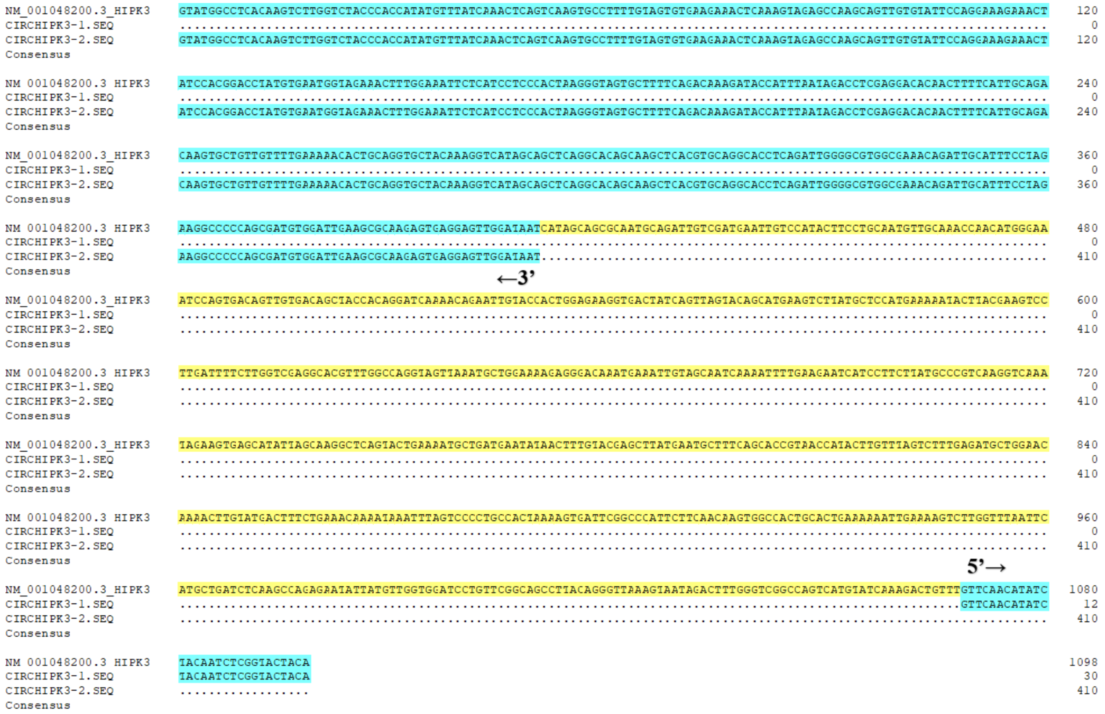

Fig. S1. CircHIPK3 plasmid clone sequence diagram.

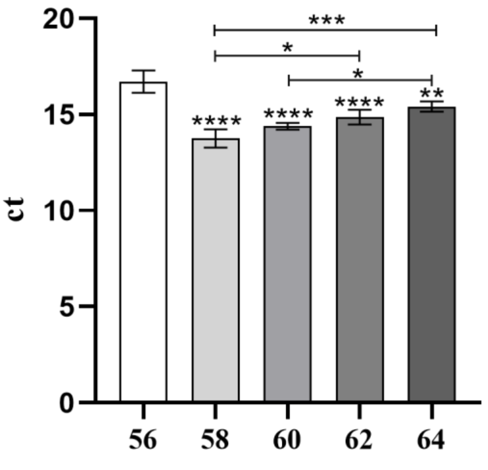

Fig. S2. PCR TM condition optimization results.

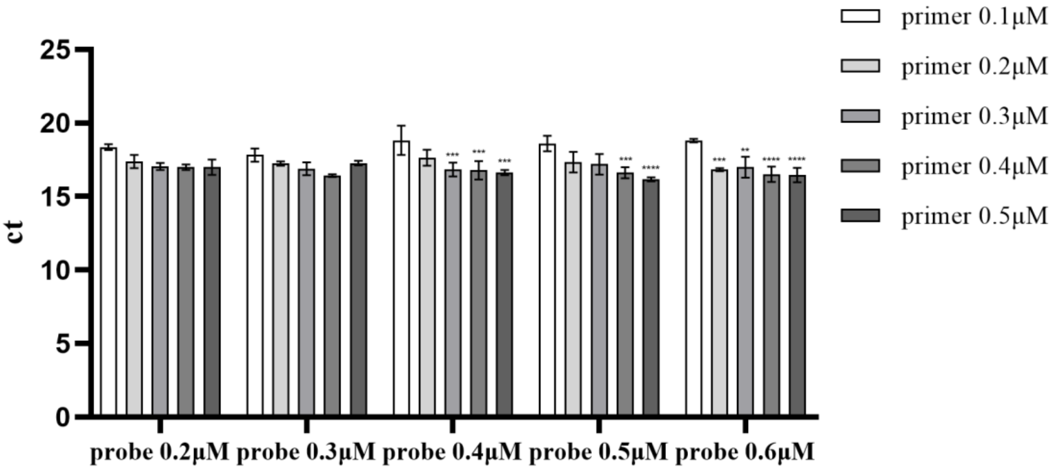

Fig. S3. Optimization results of PCR with different combination of primer and probe concentration.

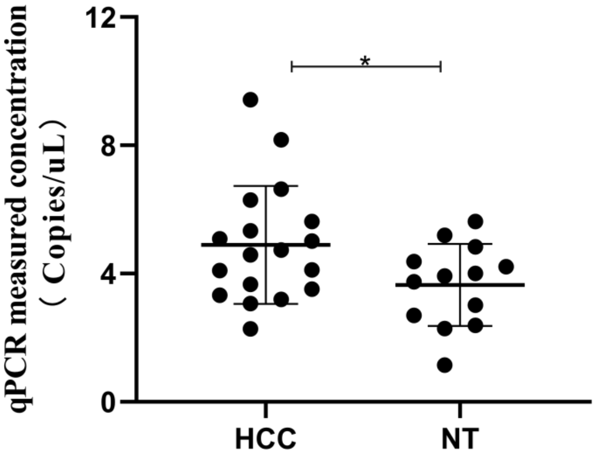

Fig. S4. qPCR results of healthy blood samples and HCC plasma samples, *P*<0.05.

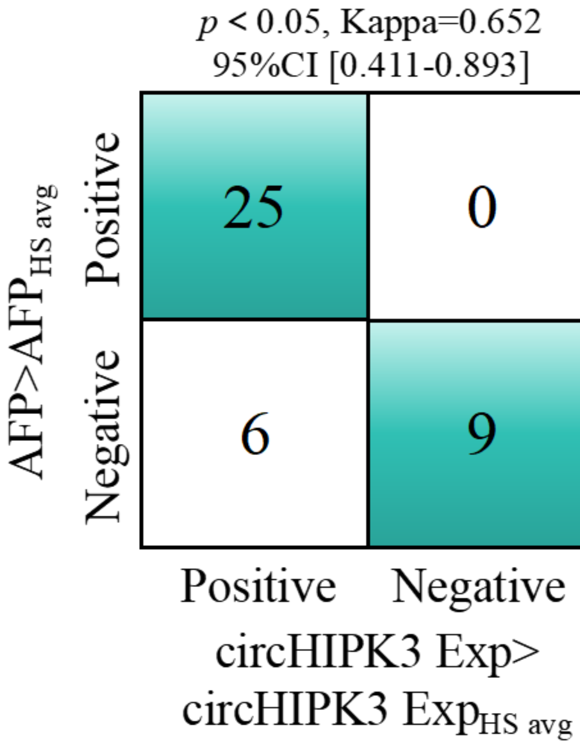

Fig. S5. Correlation analysis of AFP and plasma samples. kappa=0.652,95%CI [0.411-0.893].HS avg：Average of Healthy Samples, EXP: Expression Level.

The Approval of Ethics Committee Review

Receipt No.: SL-NBEY-KY-2023-129-01 Approval No.: YJ-NBEY-KY-2023-129-01

Study Title	Establishment and clinical evaluation of liquid biopsy method for hepatocellular carcinoma marker hsa_circ_0000284 based on ddPCR technology		
Study Source	Ningbo No.2 Hospital		
Phase Stage	Investigational Product ☐ Phase I clinical trial ☐ Phase II clinical trial ☐ Phase III clinical trial ☐Phase IV clinical trial<br>Investigational Device ☐ Class I ☐ Class II ☐ Class III<br>■ Other		
Sponsored Department	Medical laboratory department	Principal Investigator	Yuanye Ji
Review Type	Initial Review	Review Method	Expedite Review
Review Materials	■ Initial Review Application Form<br>■ Clinical Protocol (Version:V1.0, Version Date:Oct 10,2023)<br>■ Case Report Form<br>■ C.V of Principal Investigator<br>■ Team Member List<br>■ Financial Disclosure Statement<br>■ Commitment Letter<br>■ Proof of Biological Samples and Information Data<br>■ Scientific Review Opinions<br>■ Principal Investigator Responsibility Statement		
Conclusion	Agree		

Review Decision:

After reviewed by Ethics Committee (EC) according to GCP of CFDA, Ethical Review of Biomedical Research of MOST, Helsinki Declaration of World Medical Assembly, International Ethics of Biomedical Research involving human Subject of International Council of Medical Science, the decision is:

Approval

If Principal Investigator(PI) is changed or the protocol, Inform Consent Form or Recruitment Materials are revised during the study period, the amendment application should be submitted. Serious Adverse Event (SAE) should be reported promptly if any SAE is found. Processing report should be submitted to EC one month prior to the timeline of annual or scheduled review frequency required by EC. Termination or suspension report should be submitted to EC promptly if the applicant will withdraw or terminate the study. The final research project report should be submitted to EC if the study is finished.

Follow-up Review Frequency	12 months	Chop: 宁波市第二医院 人体研究伦理委员会
Approval Expiry Period	12 months	
Signature of Director of Ethics Committee	Yaoren Hu	
Date	Nov 6, 2023	
EC contact	Yanping Ren（86-574）83870361	

Figure S5: The Approval of Ethics Committee Review.

Table S1. Clinical sample information in the study.

Variable	Healthy Controls	HCC Group	p-value
Sample Size	15	25	/
Gender (M/F)	14/15(93%Male)	24/25(96%Male)	0.717
Age (years)	56.4±3.38	58.9±4.6	0.181
AFP (ng/mL)	2.6±1.2	384.3±83.8	＜0.001
